# A Randomized Controlled Trial to Manage Postoperative Ocular Pain after Pterygium Excision with Conjunctival Autograft Transplantation with a Single Application of 2% Sodium Hyaluronate

**DOI:** 10.1155/2022/5144516

**Published:** 2022-06-27

**Authors:** Winai Chaidaroon, Sirawit Isipradit, Phit Upaphong, Chutikarn Dejkriengkraikul

**Affiliations:** Department of Ophthalmology, Faculty of Medicine, Chiang Mai University, Chiang Mai 50200, Thailand

## Abstract

**Purpose:**

To assess the effectiveness of a single application of 2% sodium hyaluronate (SH) in controlling pain after pterygium excision as compared with that of a control group.

**Methods:**

We performed a prospective randomized controlled trial in the patients who underwent pterygium excision. The outcome of topical application of 2.0% SH was measured using the visual analogue scale (VAS), in comparison with that observed in a control group (without SH). The area of ocular surface defects was assessed by ImageJ freeware. Analysis of pain scores and ocular surface defects were observed from both groups immediately after the operation, Day 0, and 3 subsequent days.

**Results:**

Thirty patients were randomly divided into control group and SH treatment group. The initial area of the ocular surface defect on Day 0 was approximately the same for both groups (*p*=0.242). The medians of pain score assessed by the VAS on Days 0, 1, and 2 were 5, 3, and 0 for the SH group and 6, 5, and 3 for the control group, respectively. The pain score was statistically significantly decreased in the SH group compared to the control group on Day 1 (*p* < 0.001) and Day 2 (*p* < 0.001). The pain level of both groups was nearly the same on Day 3 (*p*=0.141). The area of ocular surface defects was significantly different between two groups on Day 1 (*p* < 0.001) and Day 2 (*p* < 0.001). Postoperative complications were not observed.

**Conclusion:**

A single topical application of 2% SH in pterygium excision was effective in relieving pain in the early postoperative period without any adverse effects. This innovation may provide alternative pain control in pterygium surgery.

## 1. Introduction

Pterygium, an ocular surface disorder, is a winged-like fibrovascular growth of the limbus and conjunctiva towards the cornea [1]. A positive correlation between the development of pterygium and ultraviolet (UV) irradiation exposure was advocated by previous studies [2, 3]. Surgical intervention is the definitive method for treating pterygium. Major indications of surgical treatment included decreased vision, cosmetic concern, chronic irritation, and ocular motility limitation [4].

Several surgical modalities have been described as methods of treating pterygium, including simple excision [5] and bare sclera excision with adjunctive therapies such as beta irradiation [6] or mitomycin C [7]. Superior conjunctival autografting for pterygium treatment is a gold standard for pterygium treatment [8] proposed by Kenyon et al. [9]. Postoperative ocular pain is the most common complaint among patients [8]. According to the prosperous innervation of the cornea and conjunctiva, any abrasion of either cornea or conjunctiva which normally occurs after pterygium surgery causes postoperative ocular pain [10, 11]. Various medications such as topical cyclopentolate [10], viscous lidocaine [11], and peribulbar morphine and lidocaine [12] are prescribed to decrease postoperative ocular pain.

Sodium hyaluronate (SH) is a polysaccharide consisting of disaccharide units of glucuronic acid and N-acetyl-glucosamine [13]. It plays a role in numerous biological functions, including cellular proliferation, anti-inflammatory effects, antioxidant protection, and wound repair [13, 14]. Several ophthalmic pharmaceutical preparations of SH such as artificial tears, eye drops, in situ-forming hydrogels, modified nanoparticles, and intravitreal injections were established to accelerate ocular surface wound healing [15]. With the rapid recovery of wound healing, the ocular pain after surgery would be speedily diminished. The effectiveness of a single application of 2% SH has not been previously evaluated for alleviating postoperative ocular pain followed by pterygium surgery. Therefore, we aim to investigate the effectiveness of a single application of 2% SH for pain control following pterygium excision and compare it with the control group.

## 2. Materials and Methods

### 2.1. Study Design

This study is a prospective randomized controlled double-blinded clinical trial, conducted for patients undergoing pterygium excision with conjunctival autograft transplantation at a single center in Thailand. Patients who were diagnosed with primary pterygium were recruited from an outpatient ophthalmology department, Chiang Mai University Hospital from September 2021 to December 2021. The study protocol was conducted in accordance with the tenets of the Declaration of Helsinki and approved by the ethics committee of Faculty of Medicine, Chiang Mai University (study code: OPT-2562-06378). The clinical trial had been registered in https://www.thaiclinicaltrials.org/ with the following identification number: TCTR 20210928002. Informed consent was obtained from all participants prior to the study. The treatment outcome of a single immediate postoperative topical application of 2.0% SH (TRB CHEMEDICA AG, Visiol®, München, Germany) was measured by postoperative pain using the visual analogue scale (VAS) [16], compared with those of the control group (without SH). Four visits for pain assessment consisted of the first visit which was immediately after the operation, Day 0. Three follow-up visits were scheduled on Days 1, 2, and 3. Simultaneously, photographs of the area of corneal and conjunctival epithelial defects after pterygium surgery were taken.

### 2.2. Study of Population and Treatment

Patients aged between 20 years and 70 years with a diagnosis of unilateral primary pterygium were eligible for the study. Patients who had recurrent pterygium, eyelid disease, ocular surface disease, ocular malignancy, ocular infections, glaucoma, concurrent ophthalmic drug use, history of previous ocular surgery or trauma, systemic diseases that interfere with corneal wound healing, dementia or mental instability, deafness, and communication barrier for VAS assessment were excluded. Patients who received regular preoperative or postoperative analgesics were also excluded from this study.

Patients were divided into two groups (the SH group and the control group). Age is a confounding factor for corneal and conjunctival wound healing that might influence pain response. They were stratified by age (<50 or ≥ 50 years old) into a permuted block of 4 randomizations using strata methods. Subsequently, the patients were randomly assigned to either the SH or the control group using 1 : 1 block randomization methods.

All patients underwent pterygium excision with superior conjunctival autografting associated with transferring a free conjunctival graft of superior bulbar conjunctiva to cover the bare sclera with an operating microscope [17]. All surgeries were done by one surgeon (W. C.) and without retrobulbar or eyelid anesthesia.

### 2.3. Surgical Procedure

1. The surgical fields included the periorbital area, eyelids, and ocular surfaces and were sterilized with 10% povidone iodine. One drop of 2% lidocaine jelly (AstraZeneca, Xylocaine Jelly 2%®, Södertälje, Sweden) was applied 10 minutes prior to the operation to induce anesthesia of the conjunctiva and cornea.
2. An eyelid speculum was applied to the eyelids to ensure adequate surgical exposure.
3. Povidone iodine and lidocaine jelly residues were rinsed from the operating eye with 0.9% of normal saline solution.
4. One mL of 1% lidocaine/1 : 100,000 adrenaline mixture (AstraZeneca, Xylocaine 1%®, Södertälje, Sweden) was injected into the subtenon space beneath the pterygium via a 27-gauze needle.
5. The head of pterygium and fibrovascular tissue extending from the cornea to the limbus were superficially excised by a surgical blade number 15. Westcott scissors were used to remove pterygium from the surrounding conjunctiva. All pterygium tissue was excised. The remaining subtenon tissue was extensively removed with minimal cauterization.
6. The patient was told to look down to expose the vivid superior conjunctiva. One percent plain lidocaine (AstraZeneca, Xylocaine 1%®, Södertälje, Sweden) of 0.5 mL was injected with a 27-gauze needle subconjunctivally between the conjunctiva and tenon at the superior border of the planned excised conjunctiva graft in the 12 o'clock position.
7. A free conjunctival graft was excised using Westcott scissors in the extract size of the bare scleral area. After placing the conjunctival graft on the bare scleral area, the graft was sutured with 9 interrupted style stitches of 8–0 polyglactin.
8. Corneal epithelial defects caused by pterygium excision and conjunctival epithelial defects at the donor site were stained by fluorescein paper strip (Chona Surgical Co., Fluorescein Sodium -Test Strip®, Delhi, India) and photographed using the digital camera equipped with a SL-D701 slit lamp (Topcon Corporation, Topcon®, Tokyo, Japan) with the cobalt blue exciter filter to record the area of the epithelial defect in both the groups.
9. A thin coat of 2% SH was applied topically to the stained-positive epithelial defect in the SH group but not in the control group by 1 investigator (S. I.) [18].
10. The eye was patched and postoperative medications were prescribed. These medications included topical 0.5% levofloxacin (Santen, Cravit®, Osaka, Japan) eye drops and 1% prednisolone acetate (Allergan, Pred-Forte®, Westport, Ireland) eye drops to be applied 4 times daily for one month in both the groups. Patients in both the groups were instructed to start using the eye drops on Day 1.

The operating time was approximately 20 minutes in all cases. If patients needed additional anesthesia due to severe intraoperative or postoperative pain, they were treated appropriately and excluded from the research protocol.

### 2.4. The Assessment of Postoperative Ocular Pain

**Figure 1:**
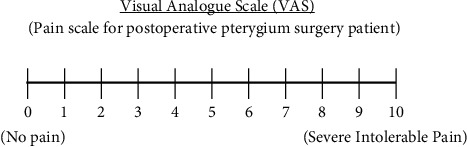
VAS (visual analogue scale) for pain assessment after pterygium excision.

The VAS was used to assess the degree of ocular pain following a surgery. Before starting the protocol, patients were advised about the VAS and were given instructions by a single investigator (S. I.) on how to assess pain. The protocol involved four assessment visits; the first visit was immediately after the operation on Day 0. Three follow-up visits were scheduled on Days 1, 2, and 3. The numerical value of VAS was determined as the length in centimeters of the patient's mark on a scale between zero (no pain) and 10 (severe intolerable pain) (Figure 1). Patients were scheduled for four complete ophthalmic examinations and pain score assessments at the same time on consecutive days. During each visit of every patient, the investigator recorded the subjective pain score measured from marks on a standard printout of the same VAS file.

### 2.5. Assessments of Conjunctival and Corneal Epithelial Defects

**Figure 2:**
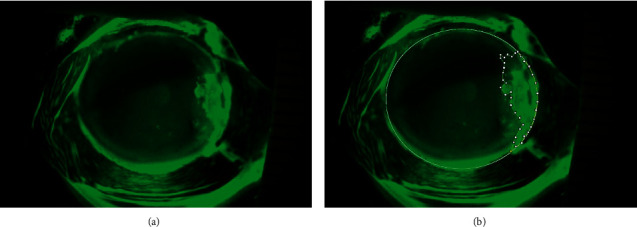
Photographs of the fluorescein-stained positive epithelial defect of the cornea after pterygium excision. (a) Original photo. (b) Photo after manual delineation of the fluorescein-stained positive corneal epithelial defect using ImageJ freeware.

**Figure 3:**
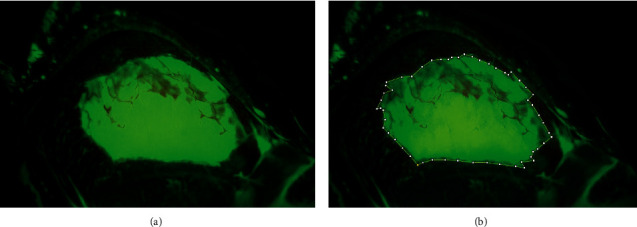
Photographs of the fluorescein-stained positive epithelial defect of conjunctiva after pterygium excision. (a) Original photo. (b) Photo after manual delineation of the fluorescein stained-positive conjunctival epithelial defect using ImageJ freeware.

The photos of both fluorescein-stained corneal and epithelial defects taken immediately after the surgery on Day 0 were saved as uncompressed Joint Photographic Experts Group files (6208 × 2294 pixels) and RGB files using the IMAGEnet®6, version 3.0.1. Patients were scheduled to visit for ophthalmic examination and daily photograph at a fixed time for three days (Day 1, 2, and 3). They were evaluated by 1 investigator (P. U.) to determine the area of corneal and conjunctival epithelial defects using ImageJ freeware 1.53 k to delineate the defect (Figures 2(a) and 2(b) and Figures 3(a) and 3(b)) [19]. The size of corneal and conjunctival epithelial defects was calculated by using ImageJ freeware.

### 2.6. Statistical Analysis

This study was designed as a prospective randomized control trial. A sample size of 30 eyes in this prospective study was calculated based on the mean pain score in the study of Oksuz H and Tamer C [11]. Analysis was performed using the SPSS 21.0 statistical software (SPSS Inc., Chicago, IL, USA). Mean (standard deviation (SD)), *t*-test, and Chi-square test were analyzed based on the demographic and clinical characteristics of participants at baseline. The comparison of pain scores between the SH group and the control group was carried out using the Mann–Whitney *U* test and median (range). The *t*-test was utilized to compare the area of corneal and conjunctival epithelial defects between the two groups. Statistical significance was considered as a *p* value less than 0.05.

## 3. Results

**Figure 4:**
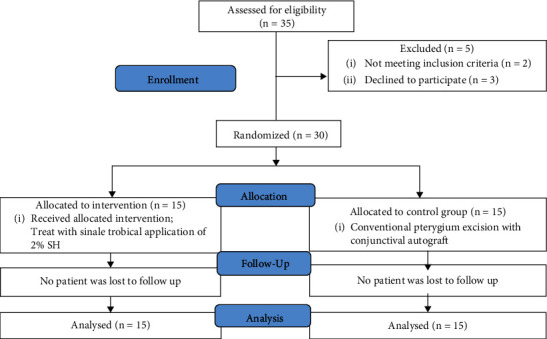
CONSORT flow diagram and follow-up of a randomized controlled trial of the SH group versus the control group for patients.

Thirty-five patients were enrolled in this study. Five patients were excluded because three patients declined to participate in the protocol, one patient had rheumatoid arthritis, and another had diabetes mellitus. Therefore, 30 patients were randomized into one of the two groups, resulting in the SH group of 15 patients in total and 15 patients in the control group. The CONSORT flow diagram is illustrated in Figure 4.

### 3.1. Baseline Characteristics

The baseline characteristics of the study patients included in the analysis are summarized in Table 1. Thirty patients, including 19 females and 11 males, were enrolled in the study. There were no statistically significant differences in the baselines of age, sex, and area of initial conjunctival and corneal defects.

### 3.2. Pain Score Assessment and Ocular Surface Defects

**Figure 5:**
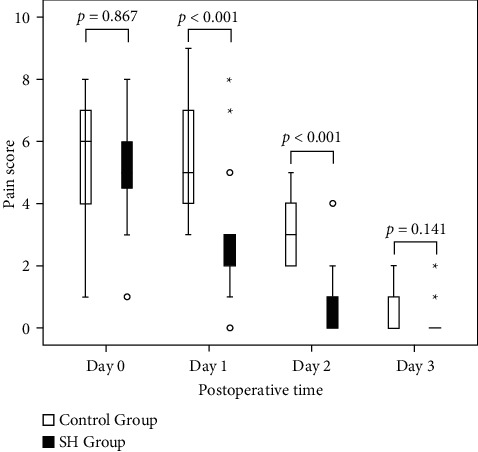
A comparison of the box plot of pain scores of the SH and the control groups on Days 0, 1, 2, and 3. The boxes represent the interquartile range (IQR). Medians are indicated by a thick horizontal line inside the box. The distribution of medians is shown by the thick horizontal lines outside the box. Outliers are depicted by individual circles and stars.

**Figure 6:**
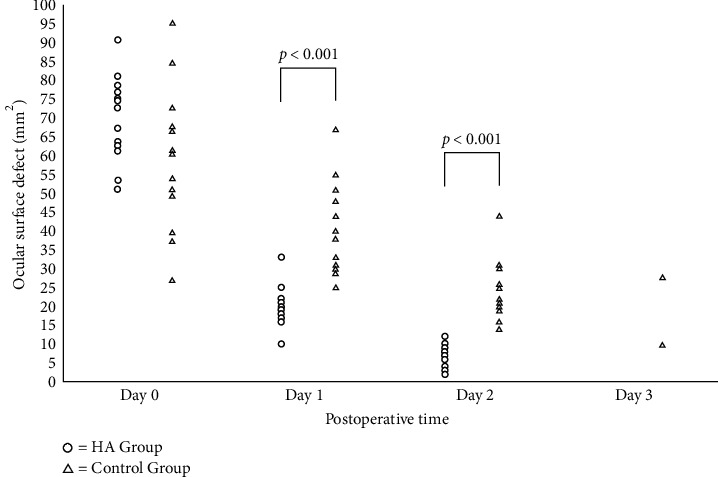
Area of ocular surface defect on Days 0, 1, 2, and 3 between the SH and the control groups.

Pain scores are summarized in Table 2. No patients in either the SH group or control group reported a pain score of zero at the baseline (Day 0). Severe intolerable pain (pain score of 10) was also not found throughout this study. The pain score assessed by VAS was statistically significantly decreased in the SH group compared to the control group on Day 1 (*p* < 0.001) and Day 2 (*p* < 0.001). On postoperative Day 3, 6.7% (1/15) of the patients in each group had a small corneal defect. However, these two patients had complete epithelialization by Day 4.  Figure 5 illustrates the pain score on Days 0, 1, 2, and 3 between the SH and the control groups by box plot.  Figure 6 shows the area of ocular surface defect on Days 0, 1, 2, and 3 between the SH and the control groups. The ocular surface defect healing, in terms of the area of ocular surface defect, was significantly different between two groups on Day 1 (*p* < 0.001) and Day 2 (*p* < 0.001). It showed that pain and ocular surface defects were decreased significantly on Day 1 and Day 2.

### 3.3. Safety Profiles

No serious adverse events were detected in the SH and control groups.

## 4. Discussion

The ocular surface, including the conjunctiva and cornea, is densely supplied by corneal sensory nerves predominantly from the ophthalmic division of the trigeminal nerve [20], making the cornea the most densely innervated tissue of the human body [21]. Any external stimuli on to the ocular surface, such as surgical intervention related to corneal or conjunctival abrasion [8, 10, 11] or photorefractive keratectomy [22], can cause a significant postoperative ocular pain. All patients in the current study had both corneal and conjunctival abrasion following pterygium excision with conjunctival autograft transplantation. They experienced postoperative ocular pain as a result of corneal and conjunctival epithelial defects.

Ocular pain is one of the most common postoperative complaints following pterygium excision [10]. Adequate pain management hastens patients' visual rehabilitation, improves satisfaction, and decreases devastating complications, such as infectious corneal ulcer and corneal perforation. Thus, a variety of modalities for pain rescue after pterygium excision was proposed. Topical cyclopentolate, a cycloplegic drug, was prescribed to relieve cyclospasm related to corneal abrasions without the contribution of the corneal nerve [23]. One study done by Goktas concluded that topical cyclopentolate is effective and well tolerated for pain control after pterygium excision; however, topical cyclopentolate could induce acute angle closure glaucoma attack, in particular, in a shallow anterior chamber patient, and reduce postoperative visual acuity as a mydriatic effect of this agent [10]. Another study demonstrated the beneficial effects of viscous lidocaine for pain relief during a short postoperative period [11]. According to gel-like properties, it may act as a barrier and reduce the effectiveness of postoperative antibiotic eye drops, potentially significantly increasing the risk of acute postoperative infection [24]. The anesthetic cornea and conjunctiva caused by the anesthetic agents are susceptible to further unintentional ocular surface abrasion. Topical nonsteroidal anti-inflammatory drugs (NSAIDs) were used to treat postoperative eye pain [25]. The mechanism of analgesic action of NSAIDs has been clearly explained on the basis of their inhibition of cyclooxygenase enzyme which synthesizes prostaglandins. Adverse events such as corneal melting resulting from topical NSAIDs were reported [26].

SH is a natural high molecular weight and linear polysaccharide with several hydrophilic functional groups. The biological function of high-molecular-weight (more than 10^6^ Da) SH is inhibition of inflammation by its interaction with the cluster of differentiation 44 cell surface receptor and the SH-mediated motility receptor [27]. The anti-inflammation, antioxidant, and cell proliferation properties of SH could promote and hasten epithelial wound healing. Nishida T et al. proposed that SH accelerated the reepithelialization in animal models [28]. Lin T et al. also supported Nishida's study with the evidence of rapid corneal epithelial healing following corneal abrasion caused by mechanical damage in Chinese patients [29]. Pain following pterygium excision with conjunctival autograft transplantation can result not only in corneal and conjunctival abrasions [10, 11] but also from inflammatory process nociceptor [30]. Thus, the rapid wound healing and the reduction of inflammation induced by surgery would decrease postoperative ocular pain.

In the current study, a single application of 2% SH, which had a high molecular weight of 1.8 × 10^6^ Da, was applied for pain following pterygium excision and compared with the control group. The results revealed that pain scores assessed by VAS were statistically significantly lower in the SH group than those in the control group on Day 1 and Day 2. The pain score and ocular surface defect were decreased significantly on Day 1 and Day 2. In addition, almost all of the patients in both groups had completely healed epithelium on Day 3 as demonstrated by the ImageJ photographs. The VAS is used to quantify pain severity. This is a continuous outcome measurement composed of a 100 mm scale from 1 to 10 cm with low and high parameters for pain-free effects and severe pain. The VAS is a practical approach and is a reliable and valid measure of acute pain [31]. This assessment is appropriate for acute ocular pain resulting from surgery of the pterygium. Another pain scale, the Wong–Baker FACES Pain Rating Scale, contained several facial expression images which were a popular method for pain measurement in children. FACES scales use facial expressions from score 0 (smiling face) to score 10 (crying face) [32]. The VAS pain measurement scale was used to assess the pain in this study instead of the Wong–Baker FACES Pain Rating Scale because the pain scale facial expressions represent how much patients feel internally, rather than how their face looks externally. Furthermore, patients had difficulty selecting facial expressions that were too similar, even though they would score differently.

For safety considerations, no serious adverse events related to 2% SH were identified in both SH and the control groups. According to the mucoadhesive effect of SH which is easily bound to the epithelial mucosa and 2% SH used in this study, there may be reasons for uncomfortable feeling in the eye [33]. However, a single application of high concentration of SH in this study did not lead to any increase in patient dissatisfaction due to matted eyelashes or decreased vision [33, 34]. Eventually, the epithelium had complete reepithelialization on Day 4 without any complication in either group. While this study demonstrated favorable results in short-term pain relief following pterygium surgery, cost-effectiveness analysis should be considered in future clinical practice.

The initial area of conjunctival and corneal defects in both groups was analyzed using ImageJ freeware. This method of measuring made it possible to delineate with precision the extent of the defect and to calculate the size of ocular surface defect with precision. Therefore, ImageJ freeware is a very useful tool for determining whether a statistically significant difference in the baseline data of the two groups.

### 4.1. Study Limitations

First, although the number of patients in this study determined a statistically difference in the results, a larger sample and longer follow-up would provide more precise measurement of those differences. Second, reactive oxidative species (ROS) were induced by UV irradiation in a pterygium patient [35]. Visiol® (2.0% SH containing mannitol) used in this study may not represent a pure 2% SH. A free-radical scavenger property of mannitol can reduce the biodegradation of SH long chains by ROS. This positive effect could prolong the length of contact between the SH and the ocular surface [36]. As a result, 2% SH containing mannitol may have a potential effect to accelerate epithelialization of the conjunctiva and cornea. Third, the pressure of eye patching immediately after operation may not be consistent which may affect ocular pain interpretation on Day 1. Finally, the study lacked a control group using regular high-viscosity artificial tears.

## 5. Conclusions

A single topical application of 2% SH in pterygium excision was effective for pain relief in the early postoperative period without any adverse effects. This innovation may provide alternative pain control in pterygium surgery. However, the cost-effectiveness analysis is an essential consideration for clinical application.

## Figures and Tables

**Table 1:** Demographic and baseline clinical characteristics of patients.

	SH group (*n* = 15)	Control group (*n* = 15)	*p* value
Age (year) (mean ± SD)	51.07 ± 8.80	50.73 ± 9.14	0.415^a^
Sex (*n*) (%)			
Male	Male = 5 (33%)	Male = 6 (40%)	0.705^b^
Female	Female = 10 (67%)	Female = 9 (60%)	
Area of initial conjunctival and corneal defects (mm^2^) (mean ± SD)	69.76 ± 10.70	60.50 ± 21.13	0.242^a^

*n* = number; SD = standard deviation; mm = millimeter; ^a^*t*-test; ^b^Chi-square test.

**Table 2:** The medians of pain score assessed by VAS immediately after operation (Day 0) and on Days 1, 2, and 3 between the SH and the control groups.

Postoperative time (day)	SH (*n* = 15)	Control (*n* = 15)	*p* value^*∗*^
0	5	6	0.867
1	3	5	0.001
2	0	3	0.001
3	0	0	0.141

*n* = number; SH = sodium hyaluronate; ^*∗*^Mann–Whitney *U* test.

## Data Availability

The data used to support the findings of this study are included within the article. Further data or information is available from the corresponding author upon request.
